# Ultra-thin Ag/Si heterojunction hot-carrier photovoltaic conversion Schottky devices for harvesting solar energy at wavelength above 1.1 µm

**DOI:** 10.1038/s41598-023-31982-1

**Published:** 2023-04-03

**Authors:** Zih-Chun Su, Chung-Han Chang, Jia-Ci Jhou, Hsin-Ting Lin, Ching-Fuh Lin

**Affiliations:** 1 Graduate Institute of Photonics and Optoelectronics, National Taiwan University, No. 1, Sec. 4, Roosevelt Road, Taipei, 106319 Taiwan; 2 Graduate Institute of Advance Technology, National Taiwan University, No. 1, Sec. 4, Roosevelt Road, Taipei, 106319 Taiwan; 3 Graduate Institute of Electronics Engineering, National Taiwan University, No. 1, Sec. 4, Roosevelt Road, Taipei, 106319 Taiwan; 4 Department of Electrical Engineering, National Taiwan University, No. 1, Sec. 4, Roosevelt Road, Taipei, 106319 Taiwan

**Keywords:** Photovoltaics, Solar energy and photovoltaic technology

## Abstract

Traditional silicon solar cells can only absorb the solar spectrum at wavelengths below 1.1 μm. Here we proposed a breakthrough in harvesting solar energy below Si bandgap through conversion of hot carriers generated in the metal into a current using an energy barrier at the metal–semiconductor junction. Under appropriate conditions, the photo-excited hot carriers can quickly pass through the energy barrier and lead to photocurrent, maximizing the use of excitation energy and reducing waste heat consumption. Compared with conventional silicon solar cells, hot-carrier photovoltaic conversion Schottky device has better absorption and conversion efficiency for an infrared regime above 1.1 μm, expands the absorption wavelength range of silicon-based solar cells, makes more effective use of the entire solar spectrum, and further improves the photovoltaic performance of metal–silicon interface components by controlling the evaporation rate, deposition thickness, and annealing temperature of the metal layer. Finally, the conversion efficiency 3.316% is achieved under the infrared regime with a wavelength of more than 1100 nm and an irradiance of 13.85 mW/cm^2^.

## Introduction

For nearly half a century, the greenhouse effect has led to a more frequent extreme climate. In the face of environmental crisis, it is necessary and urgent to develop renewable and low-pollution alternative energy in order to replace the traditional coal-fired power plant. Therefore, the most abundant and widely available solar energy on the earth has become one of the most potential candidates. Because the silicon resource is abundant in the earth, which is the second-largest element after oxygen, taking silicon as solar-cell material has great advantages in cost. The manufacturing technology and related equipment of silicon crystal solar cells have been relatively mature and have the advantages of good photoelectric-conversion efficiency and long-term stable operation^1–5^. In 2020, the market share of silicon crystal solar cells is still nearly 90%. So far, the photoelectric-conversion devices developed in the laboratory have reached 26.7%^6^. However, due to the limitation of its own material energy bandgap (1.12 eV), the range that silicon crystal solar cells can absorb is mostly 300–1100 nm visible light and some near-infrared light, accounting for 80.43% of the radiation energy of the whole AM 1.5G solar spectrum, whereas the remaining energy (i.e., near-infrared light above 1100 nm to medium-infrared light) accounts for 19.57%, which cannot be effectively utilized^7–9^. To make effective use of sunlight with a wavelength of more than 1100 nm, a light-absorbing layer with an energy gap of less than 1.1 eV was added to the multi-junction stack. In the past, compound semiconductors were mostly used, such as GaSb, InAs, CIS, or InGaAsSb compounds. These materials have matured epitaxial technology and are often used in high-efficiency tandem solar cells. At present, the efficiency of the 6-junction tandem structure developed in the laboratory has reached 47.1%^10^. Although the multi-junction tandem cells made of III or V group materials show excellent conversion efficiency, due to the low stock of raw materials on the earth, which has relatively high cost of materials, and limited to the small area epitaxial growth technique, coupled with the complexity of the manufacturing process and high environmental requirements, it is not conducive to large-scale production like Si solar cells and is difficult to transit to the commercialization at present.

In addition to compound semiconductors with a low-energy gap, the material that can absorb infrared light above 1100 nm is metal^11^, and almost all metals can absorb infrared light. From the principle of optically- or plasmon-induced hot-carrier science and technology^12^, the illumination of the metal–semiconductor structure sets off a cascade of complex processes^13,14^ and the energy of incident photons able to excite high-energy hot electrons under infrared irradiation have also been demonstrated^15,16^. Typically, hot carriers in metal/silicon structures can be generated in two ways, either by free-carrier absorption or by plasmon decay^17^. In the free-carrier absorption process, the energy of the hot carrier is excited from the orbital domain of the metal. After the hot carrier is generated, the remaining kinetic energy can reach another material by overcoming the work function, or it can move to another material through the built-in electric field formed by the energy-order mismatch between the metal and the other material^18^. In the plasmon decay process, the energy of the plasma is given to the hot carrier. Compared to free-carrier absorption, the hot carriers generated by plasma decay are energetic^13,18^. Using the metal film as the infrared light–absorption layer, when the electron sea in metal absorbs the solar energy, the hot carrier subgroup is excited and similar to the solar spectrum—Boltzmann energy distribution^19^, and these hot carriers are derived and collected into a photocurrent. However, there are still challenges using metal as the absorption layer. The lifetime of hot carriers in metal is very short, and due to the lack of an energy gap of metal, the excited hot carriers interact with other carriers or phonons to redistribute energy^20^, and the electrons transiting to the high-energy level will fall back to the low-energy level^21^ less than a picosecond, making it difficult to collect the hot carrier energy.

A possible route to overcome the short-lifetime issue is to form a Schottky barrier with appropriate semiconductor–metal contact to capture hot carriers. Hot carriers with energy higher than the Schottky barrier can be injected into semiconductors. The energy required for the hot carrier to cross the energy barrier in this system is much smaller than the semiconductor bandgap^22,23^. This photovoltaic system has the opportunity to improve the absorption of the infrared spectrum. In the realization of the metal plasmonic photovoltaic system, in 1996, Zhao et al.^24^ published research showing that a photocurrent is generated by visible-light irradiation on TiO_2_ electrodes with a coating of gold or silver nanoparticles. This is because the wide bandgap (3.3 eV) of TiO_2_ means that the source of a photocurrent is not generated by the TiO_2_ electrode, which indirectly proves the feasibility of generating a photocurrent through metal absorption. In 2017, the Raphael St-Gelais team published a thermoelectric-conversion device that uses the metal layer to absorb a long wavelength^25^. Through the Schottky barrier generated by the contact between TiSi_2_ and silicon, it blocks the hot carriers crossing the barrier through the metal layer in order to avoid the energy loss caused by carrier recombination. At the same time, because the Schottky barrier is lower than the silicon energy bandgap, the cutoff wavelength is extended to 1550 nm^25^. The Schottky barrier is related to metal work function. Choosing appropriate materials can even expand the cutoff wavelength to 2000–3000 nm. In 2016, to further improve the photocurrent, Reineck and Philipp’s group^26^ published that by controlling the size and arrangement density of gold nanoparticles on the TiO_2_ substrate, properly adjusting the size of metal nanoparticles can enhance the generation, transfer efficiency, and relaxation time of hot carriers^26–28^. Some studies have focused on the time scale of hot-electron generation, injection, and regeneration mechanism. Rapid injection into adjacent semiconductors before hot carrier recombination or decay is key in improving conversion efficiency^29,30^. Furube, Du team^31–33^ used the ultrafast visible light pump/infrared probe femtosecond transient-absorption spectrum to characterize charge transfer kinetics, monitored the transient-absorption of Au/TiO_2_ at 3500 nm, and confirmed that the electron relaxation of gold nanoparticles occurs through electron–electron interaction(< 100 fs) . To further improve the energy-conversion efficiency of this process, the key point is to realize faster hot-electron injection before energy loss through electron–electron interaction.

A series of related studies have been done on testing other metal types, such as precious metal nanoparticles^34–37^, or enhancing hot carrier excitation with nanoparticles with specific geometry^38–43^. However, so far, no relevant results have been proposed for the photoelectric-conversion efficiency of an infrared regime. We propose to break through the practice of hot carrier collection and conversion into a current, use the silver-silicon interface structure, evaporate a layer of silver film with a thickness of several nanometers on the silicon substrate, make use of the infrared light–absorption characteristics of the silver film, absorb the near-infrared light with wavelength beyond 1100 nm to the middle infrared region, which cannot be used by silicon crystal, and convert it into a photocurrent so as to effectively use the whole solar spectrum.

In this study, using the most common single silicon wafer on the market, through a relatively simple and low-cost thermal evaporation process, we successfully made metal–silicon junction which called hot-carrier photovoltaic conversion Schottky device that can absorb the infrared spectrum of more than 1100 nm, breaking through the dilemma that the current commercial silicon crystal solar cells cannot effectively use the infrared band in the solar spectrum. By controlling the thickness, evaporation rate, and annealing temperature of the metal layer, the absorptivity and conductivity of the metal layer were optimized so as to improve the photocurrent output. In terms of photovoltaic performance, for the infrared spectrum with a wavelength above 1100 nm and irradiance of 13.85 mW/cm^2^, the conversion efficiency is 3.316%. This result is the first time the 3.316% conversion efficiency was revealed although their efficiency is lower than that of current commercial silicon solar cells. Conventional single-silicon p–n junction solar cells have been developed for more than 40 years to obtain a conversion efficiency of about 20%. In contrast, hot-carrier photovoltaic conversion Schottky device using the metal–semiconductor junction have just been revealed. When the perovskite solar cell started out, its conversion efficiency was only 3.8%. We hope that this result will inspire more efforts to speed up the related research so that hot carrier solar cells can reach their theoretical efficiency limit, which is 66% at one sun and 86% at the concentrated condition.

## Results and discussion

**Figure 1:**
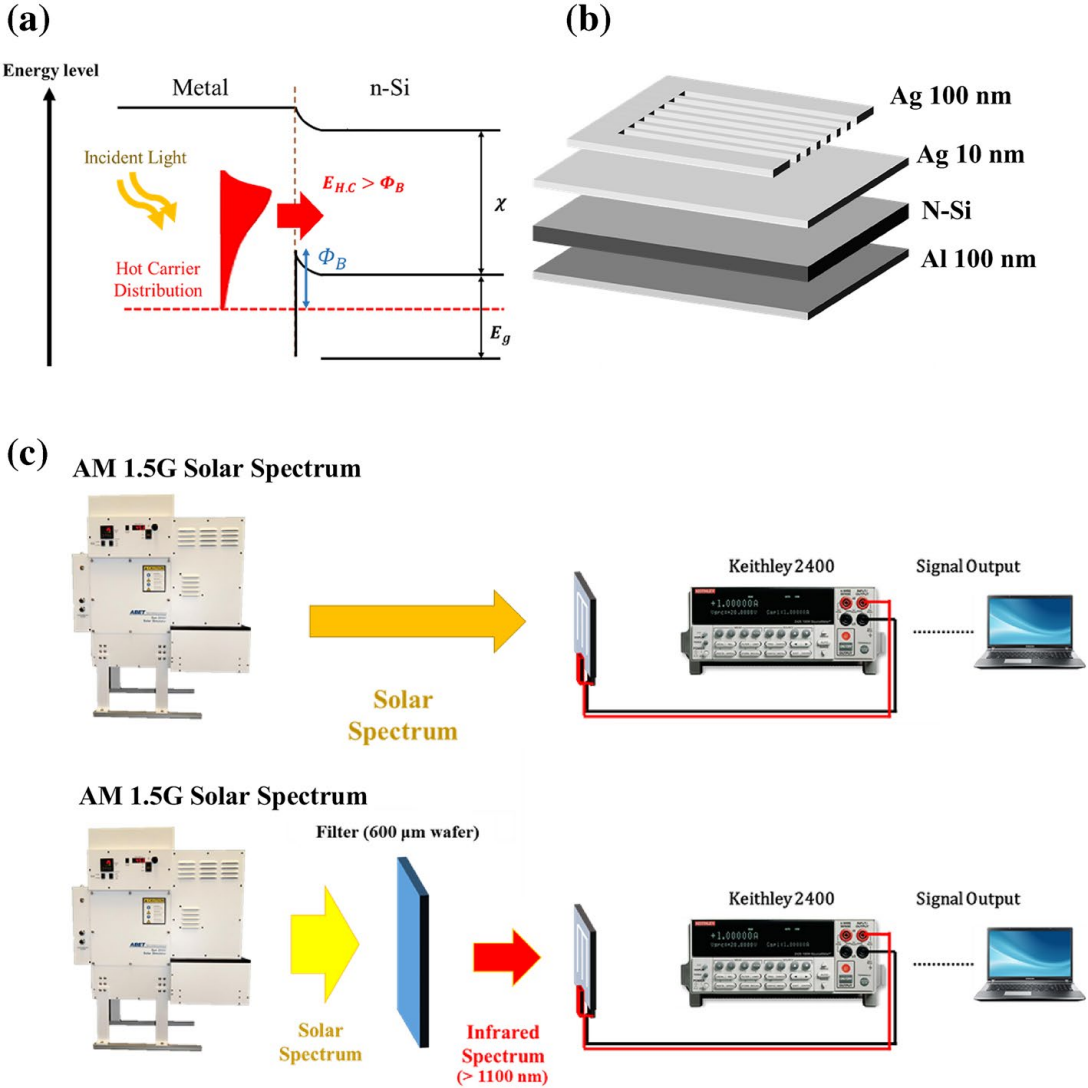
Schematic diagram of (**a**) working principle, (**b**) structure of hot-carrier photovoltaic conversion Schottky device, and (**c**) photovoltaic characteristic measurement architecture.

We developed hot-carrier photovoltaic conversion Schottky device based on the concept of hot carriers. The operation principle is shown in Fig. 1a. The silver film of nanometer thickness is used to absorb the incident spectrum, and the excited hot carriers cross the Schottky barrier and are collected by electrode to generate a photocurrent. We used the n-type silicon substrate with silver metal film; the theoretical barrier height is 0.59 eV, so it can absorb infrared light in the band below 2.1 μm. The completed structure of the hot-carrier photovoltaic conversion Schottky device is shown in Fig. 1b. The best conversion efficiency was obtained from the measurement of components with different evaporation rate parameters. With the rate of the former best results, we deposited metal layers with different thicknesses (9–10 nm) and tested the influence on metal layer appearance and solar efficiency. The distribution of silver film on the silicon substrate was observed by Scanning Electron Microscope (SEM), the surface roughness of the thin film was calculated by Atomic Force Microscope (AFM), and the optical character was analyzed by spectrophotometer.

To examine the contribution of the metal-absorption mechanism to solar efficiency, we first excluded the contribution of silicon-absorption using a measurement configuration to filter out the light with a wavelength below 1100 nm in the absorbable silicon-range, leaving only the longer wavelength regime of the AM1.5G sunlight spectrum. The measurement configuration is shown in Fig. 1c. A thick 600-μm wafer (Type: n, orientation: 1–0-0, resistance: 2–7 Ω cm, Thickness: 600–610 μm) was used as the filter, and the transmission spectrum of the silicon wafer was obtained through the analysis of the spectrophotometer (JASCOv770 spectrometer). We confirmed that it was opaque below 1100 nm and that the transmittance above 1100 nm is 55%. The penetrating light with the remained intensity measured by the Ophir Nova II P/N 7z01550 power meter was 13.85 mW/cm^2^, and this result was substituted into the incident light irradiance parameter in the conversion-efficiency formula. Based on this framework, the characteristic curve of a hot-carrier photovoltaic conversion Schottky device in an infrared regime above 1100 nm was measured, and its photoelectric conversion efficiency was calculated. The usual measurement setup using entire solar AM1.5G sunlight spectrum without the filter is also shown in Fig. 1c. Before measurement, we tested the commercially available single crystal silicon p–n junction solar cells. Without the use of filters, the conversion efficiency of the cells is 17.67%. When the filter is used, the conversion efficiency is 0.256%, almost 0. Therefore, we can determine that most of the silicon-absorption contribution can be excluded under this architecture and only the metal-absorption contribution is obtained.

All characteristic analyses were measured in an atmospheric environment. There are 2 measurement structures for the J–V characteristic curve: one is that the incident light condition is AM 1.5G and the irradiance is 100 mW/cm^2^; and the other is that the infrared regime with a wavelength above 1100 nm and the irradiance is 13.85 mW/cm^2^ after filtering. Keithley 2400 is used for measurement. The SEM uses the jeol jsm-7800f. field-emission electron microscope, and the working voltage is 10 kV. AFM was measured using the commercial scanning probe microscope multimode 3D SPM developed by the di (digital instrument) team. Vis IR spectroscopy was measured using a JASCO v770 spectrometer.

**Figure 2:**
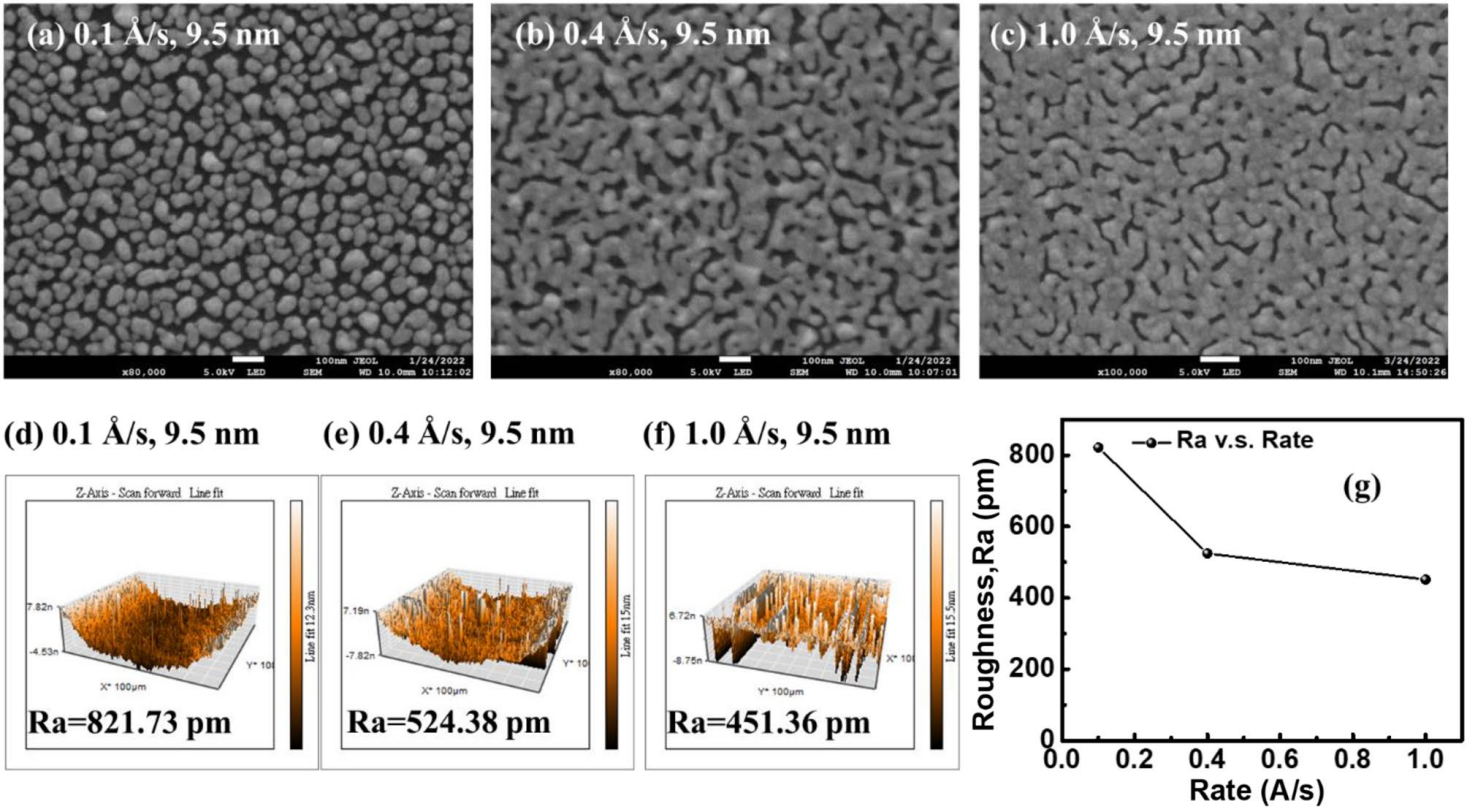
(**a**–**c**) SEM surface images and (**d**–**f**) AFM images of silver films with different evaporation rates, and (**g**) average roughness R_a_ of films versus rate.

In this study, the morphology of the metal films is the key in improving the device-conversion efficiency. We tested various evaporation rates and thickness parameters in the process of thermal evaporation to deposit a metal film onto a substrate. First, we deposited 9.5 nm of silver onto a silicon substrate with 3 evaporation rates, 0.1, 0.4, and 1.0 Å/s, respectively, and observed the distribution of silver atoms in nano scale by SEM. It can be observed from Fig. 2a–c that the silver film with a rate of 0.1 Å/s presents multiple discontinuous island structures in nano scale. With an increased evaporation rate, the distribution of silver atoms becomes more uniform, the gap between islands shrinks, and the discontinuity decreases. AFM was used to scan the surface profile of the film and calculate the average linear roughness R_a_ of the film surface. The results are shown in Fig. 2d–f, and then a variation trend with an evaporation rate could be obtained; as shown in Fig. 2g. The average roughness of the evaporation rate is 821.73 pm at 0.1 Å/s, decreases to 524.38 pm for the rate rising to 0.4 Å/s and then to 451.36 pm at 1.0 Å/s. From the SEM and AFM images, it can be determined that with the increase of the evaporation rate, the surface roughness of the film decreases and becomes smoother and more continuous. This is because increasing the evaporation rate of silver can give silver atoms greater kinetic energy, improve the mobility of silver atoms when deposit onto the substrate, and form a more uniform film structure. The gap between the islands will cause the carrier to be limited in the migration process and reduce the conductivity of the metal layer. Therefore, a high-evaporation rate is helpful to improve the carrier conduction of the device.

**Figure 3:**
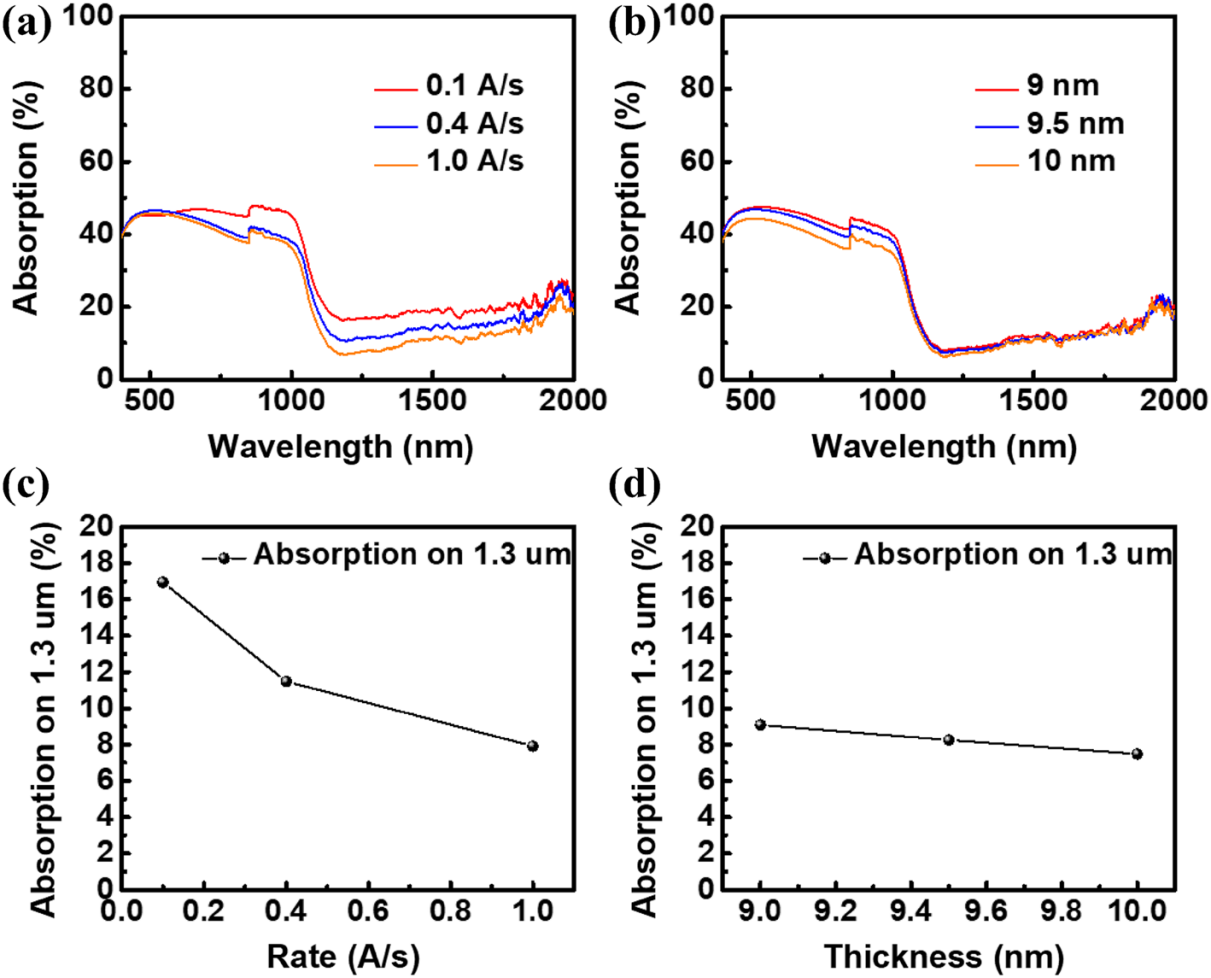
Absorptivity of silver films with different (**a**) evaporation rates and (**b**) silver film thickness deposited on silicon substrate, and (**c**,**d**) absorptivity for wavelength 1.3 μm incident light.

In terms of light absorptivity, Fig. 3a shows the spectral absorptivity of a metal–silicon junction structure with different evaporation rates. The absorptivity of the structure with an evaporation rate of 0.1 Å/s to the incident light with a wavelength of 1300 nm is 17%, the absorptivity at 0.4 Å/s is 11.5%, and the absorptivity at 1.0 Å/s is 7.9%.

Figure 3c shows that the absorption rate changes at 1300 nm. When the evaporation rate is higher, the film roughness is lower and there is less chance of incident light reflecting between the gaps of the island; also, the light-absorption rate decreases instead. Therefore, a high-evaporation rate will weaken the light-catching ability and reduce absorptivity of device. Hence, it is necessary to strike a balance between the 2 factors.

**Figure 4:**
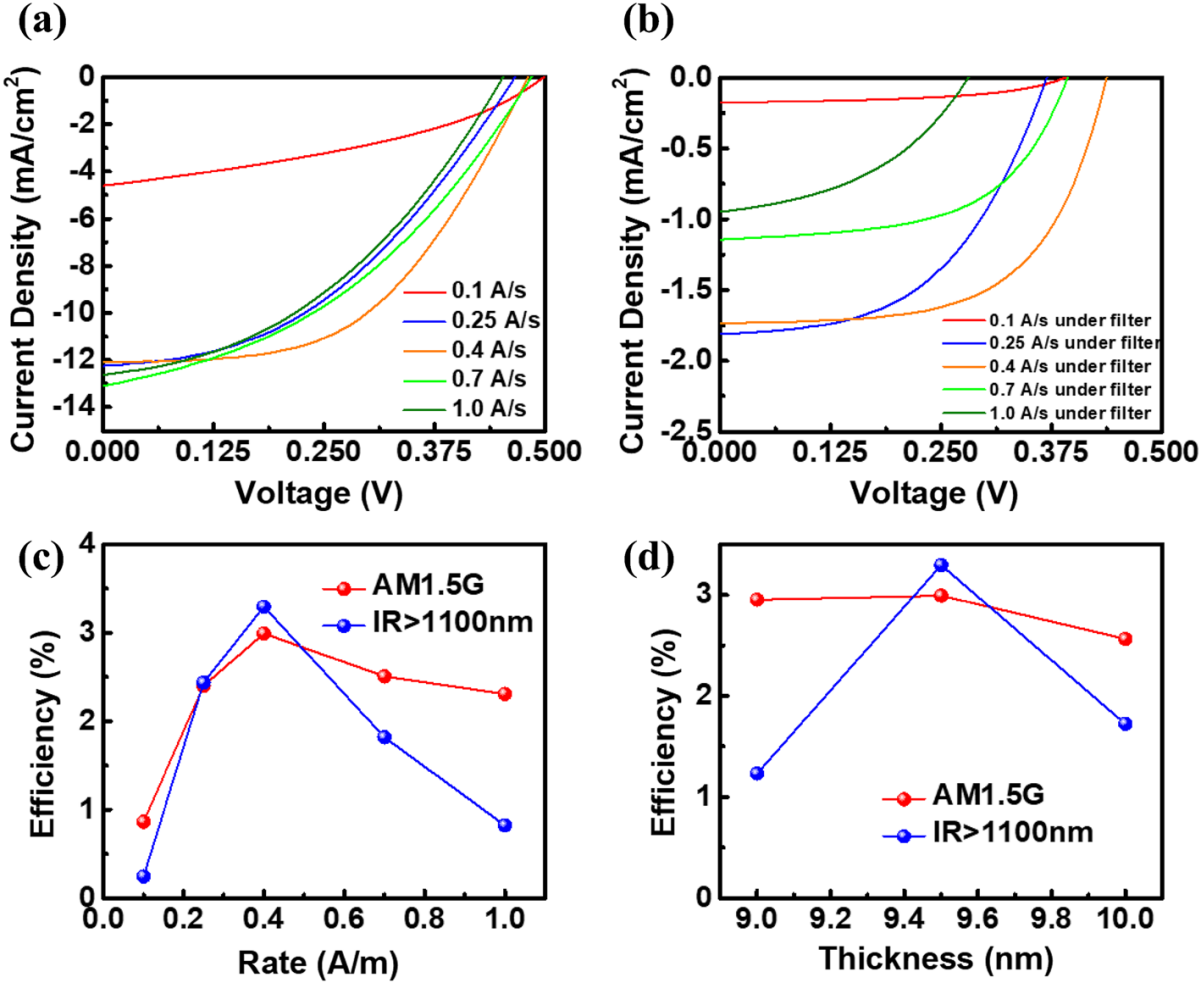
J–V curves of metal/silicon devices with different evaporation rates (**a**) without filter and (**b**) with filter, and the change trend of photovoltaic conversion efficiency against (**c**) evaporation rate and (**d**) metal film thickness.

To find the best evaporation rate conditions, we made the devices with more evaporation rates, 0.1, 0.25, 0.4, 0.7, and 1.0 Å/s, respectively, and measured the J-V curve under illumination. The results are shown in Fig. 4a. Comparing the photovoltaic characteristics with 5 evaporation rates, it can be found that the series resistance R_s_ gradually decreases with the rate increase. At 0.1 Å/s, the highest series resistance is 36.39 Ω, whereas the series resistance of 0.4 Å/s is 7.33 Ω. This is because, at a high-evaporation rate, the film is denser and more uniform and has better carrier derivation ability. In terms of conversion efficiency, the conversion efficiency of 0.864% at 0.1 Å/s is the lowest. With the rate increase, the efficiency also increases. The best conversion efficiency is 2.992%, measured at the rate of 0.4 Å/s. Then the efficiency begins to decline, and 2.308% is measured at 1.0 Å/s. Under the condition of a higher evaporation rate, the reason for the decline of efficiency is that the gap between silver film islands becomes smaller, the roughness decreases, and the resonance of incident light between the gaps becomes weaker. Therefore, the conversion efficiency is reduced. When the filter is added, under the condition that the device can only receive infrared light with a wavelength above 1100 nm and an irradiance of 13.85 mW/cm^2^, the measured current–voltage curve is shown in Fig. 4b. Devices under conditions of filtering out wavelength below 1100 nm have a minimum efficiency of 0.247% at an evaporation rate of 0.1 Å/s. With an increased evaporation rate, the conversion efficiency to infrared light increases, and the best efficiency is 3.295% when measured at 0.4 Å/s. When the evaporation rate continues to increase, even though the conductivity increases, the film gap shrinks. The resonance effect of infrared light inside the film is weakened, resulting in the decrease of the absorption rate of the film. Therefore, the efficiency is greatly reduced to 0.822% at 1.0 Å/s. All photovoltaic characteristics of hot-carrier photovoltaic conversion Schottky devices with different evaporation rates under different solar spectrum are listed in Table 1. According to the conversion efficiency mapping corresponding to the different evaporation rates, Fig. 4c can be obtained. The efficiency change trend with filtering conditions is the red line, and that with filtering conditions is the blue line. The change trend of conversion efficiency under different evaporation rates is consistent regardless of whether it is under filtering or not.

**Table 1:** The photovoltaic characteristics of hot-carrier photovoltaic conversion Schottky devices with different evaporation rates under different solar spectrum.

Evaporation rate	Voc (V)	Jsc (mA/cm^2^)	Fill factor (%)	Solar eff. (%)
Without filter				
0.1 Å/s	0.498	4.60	37.68	0.864
0.25 Å/s	0.466	12.24	42.06	2.400
0.4 Å/s	0.480	12.09	51.49	2.992
0.7 Å/s	0.485	13.09	39.51	2.506
1.0 Å/s	0.452	12.62	40.41	2.308
With filter				
0.1 Å/s	0.390	0.175	50.18	0.247
0.25 Å/s	0.369	1.813	50.38	2.436
0.4 Å/s	0.438	1.734	60.17	3.295
0.7 Å/s	0.393	1.144	56.03	1.819
1.0 Å/s	0.281	0.946	42.87	0.822

**Figure 5:**
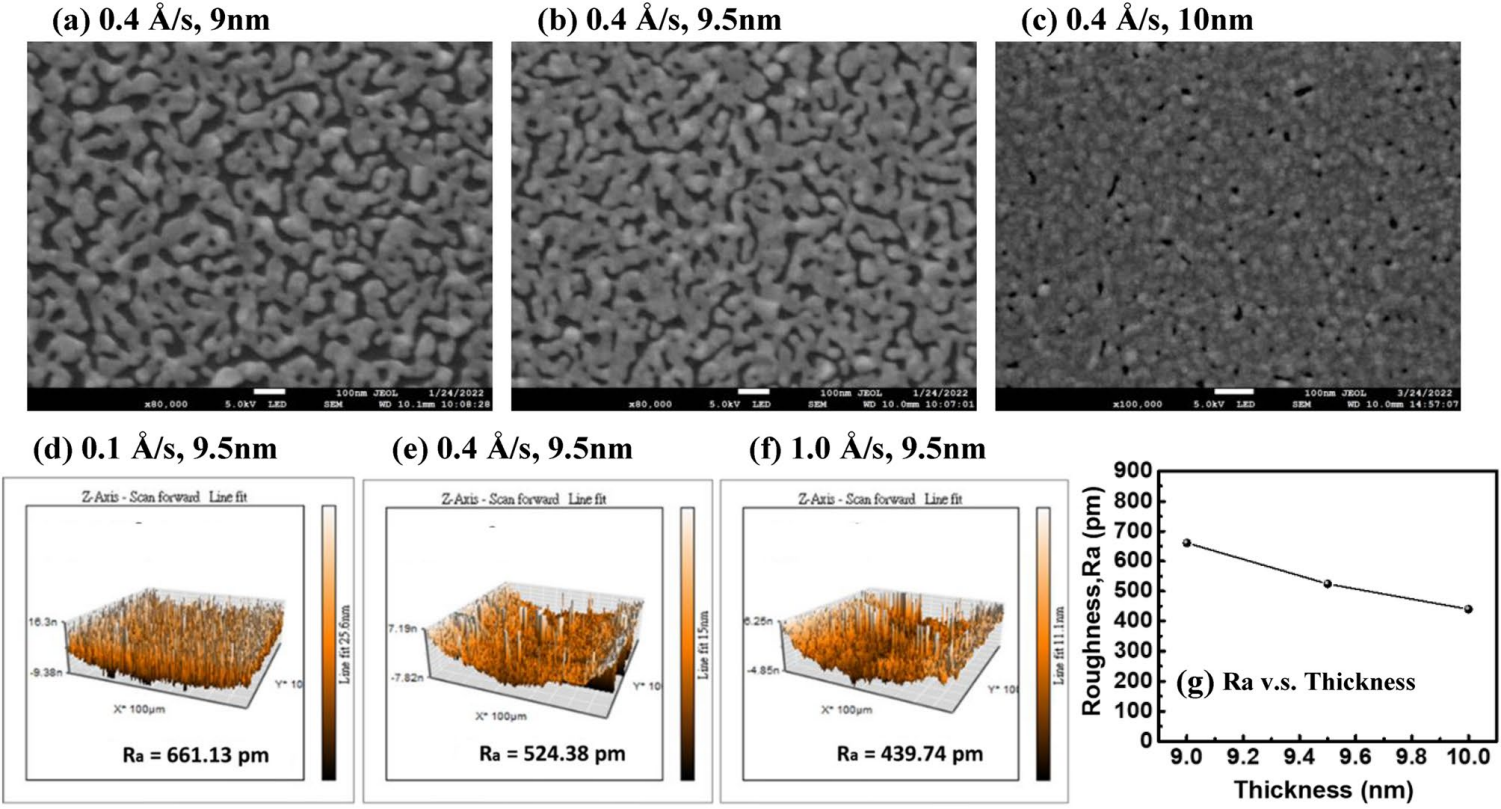
(**a**–**c**) SEM surface images and (**d**–**f**) AFM images of silver films with different metal film thickness, and (**g**) average roughness R_a_ of films versus thickness.

From the above experimental results, we determined that the highest conversion efficiency can be obtained by depositing silver films at the rate of 0.4 Å/s. Therefore, the characteristics of silver films with different thickness were tested at the evaporation rate of 0.4 Å/s. At the initial stage of the metal film, the deposition process will preferentially grow laterally to fill film gaps, as shown in Fig. 5a–c SEM images. We tested 3 thicknesses of 9 nm, 9.5 nm, and 10 nm. From the SEM, it can be observed that the gap between islands gradually shrinks with the increase of evaporation thickness. The film surface roughness R_a_ was calculated by AFM scanning as shown in Fig. 5d–f, and the variation trend of R_a_ with thickness was obtained as shown in Fig. 5g. The roughness of the 9-nm film is 661.13 pm. When the thickness increases, the roughness of 9.5 nm decreases to 524.38 pm, and the roughness of 10 nm decreases to 439.74 pm.

The SEM and AFM images show that when the film thickness increases, the size of gap between islands becomes smaller and more continuously, which is helpful to transport carriers. However, the decrease of film roughness causes the resonance effect of incident light between the film gaps to become weaker. Figure 3b shows the measurement results of films’ absorptivity with different thickness. Figure 3d shows that the absorptivity at 1300 nm is decreasing, so it is necessary to find a balance between the 2 mechanisms and to determine the best metal film type. Silver films with different thicknesses are made into devices. In the same way as the measurement framework for the change of evaporation rates discussed above, the conversion efficiencies of different metal layer thicknesses were measured respectively without a filter. The red line in Fig. 4d was obtained according to the measurement results of solar efficiency corresponding to the thickness parameters. When the metal film thickness increases from 9 nm to 9.5 nm, the efficiency increases from 2.953% to 2.992%, which is due to the improvement of the conductivity of the film. When it increases from 9.5 nm to 10 nm, the light absorption of the film decreases and the efficiency decreases to 2.564%, and the maximum value is found at 9.5 nm. The solar efficiency measured above is the conversion efficiency of the full-band solar spectrum, including the contribution of silicon absorption and metal absorption. To examine the metal absorption and conversion contribution of the device, the filter is used to cover the device surface, and only infrared light above 1100 nm is allowed to pass through. The infrared light–conversion efficiency measured under this framework is 13.85 mW/cm^2^. The measurement results are represented as the blue line in Fig. 4d, which is the same as the efficiency trend under full-spectrum irradiation. When the thickness is 9.5 nm, the maximum conversion efficiency for the infrared regime above 1100 nm is 3.295%.

**Figure 6:**
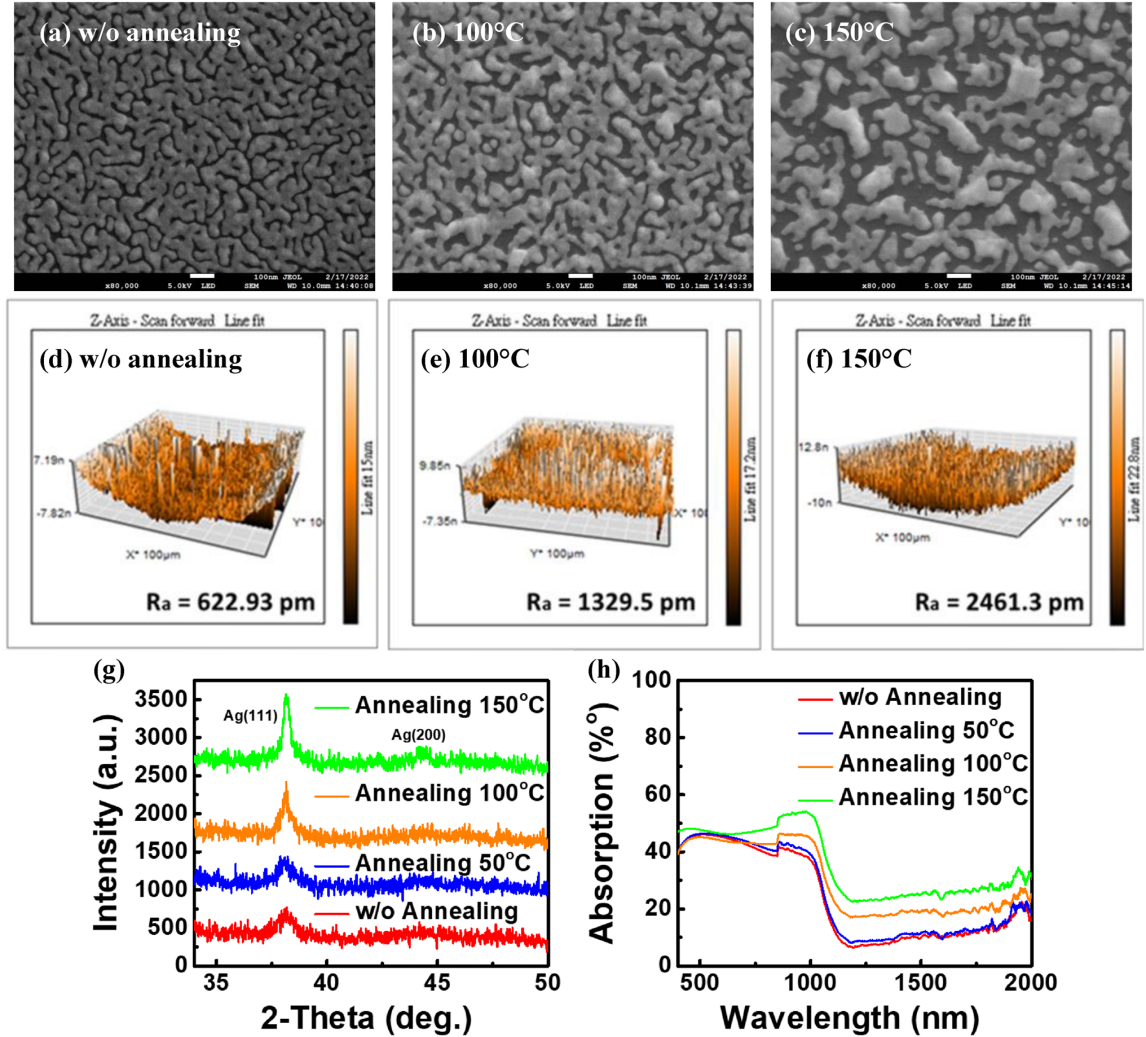
(**a**–**c**) Surface SEM, (**d**–**f**) AFM figures, (**g**) XRD pattern and (**h**) absorptivity of silver films with 9.5 nm thickness at different post-annealing temperatures.

Post-annealing is often used to improve hot-carrier photovoltaic conversion Schottky device’s electrodes to retain the high conductivity of metals and create metallic nanostructures for enhancing its optical and plasma performance. Therefore, we post-annealed the hot-carrier photovoltaic conversion Schottky devices at different temperatures to hopefully reduce the interface impedance between the metal film and the substrate, optimize the crystallization of the metal film, and improve transportation of hot carriers. The conditions are as follows. Silver films made with an evaporation rate of 0.4 Å/s and a thickness of 9.5 nm were annealed for 10 min at 50 °C, 100 °C and 150 °C. The silver film surface was observed using SEM and AFM. The SEM images are shown in Fig. 6a–c. The film transforms approximately at 100 °C. The small metal islands merge with each other to coarsen the grains. At 150 °C, the grain size is even larger. The gap between islands increases significantly and several isolated metal islands appear. Figure 6d–f are the AFM images of the films. The average surface roughness R_a_ increases with the increase of annealing temperature. In addition, XRD was measured to examine the diffraction peak of the silver film, as shown in Fig. 6g. The double diffraction angles at 2θ = 38.16° and 44.44° are diffraction peaks of silver crystalline phase [111] and [200], respectively. It can be observed that with the increase of post-annealing temperature, the intensity of diffraction peaks at the two angles gradually increase. It means the rearrangement of the lattice, promoting the consolidation of the grains and the increase of its crystallinity to help the carrier transport on the surface and the interface between films. The absorptivity of silver-silicon structure is shown in Fig. 6h. The absorbance increases with the increase of annealing temperature. At wavelength of 1300 nm, the absorptivity of the device without annealing is 7.5%. At 50 °C, it increases slightly to 9%. At 100 °C, the film gap increases, the absorptivity increases to 17.6% significantly, and finally increases to 23.3% at 150 °C. As the annealing temperature increases, the roughness of the silver film increases, which improves the light capture ability of the devices.

**Figure 7:**
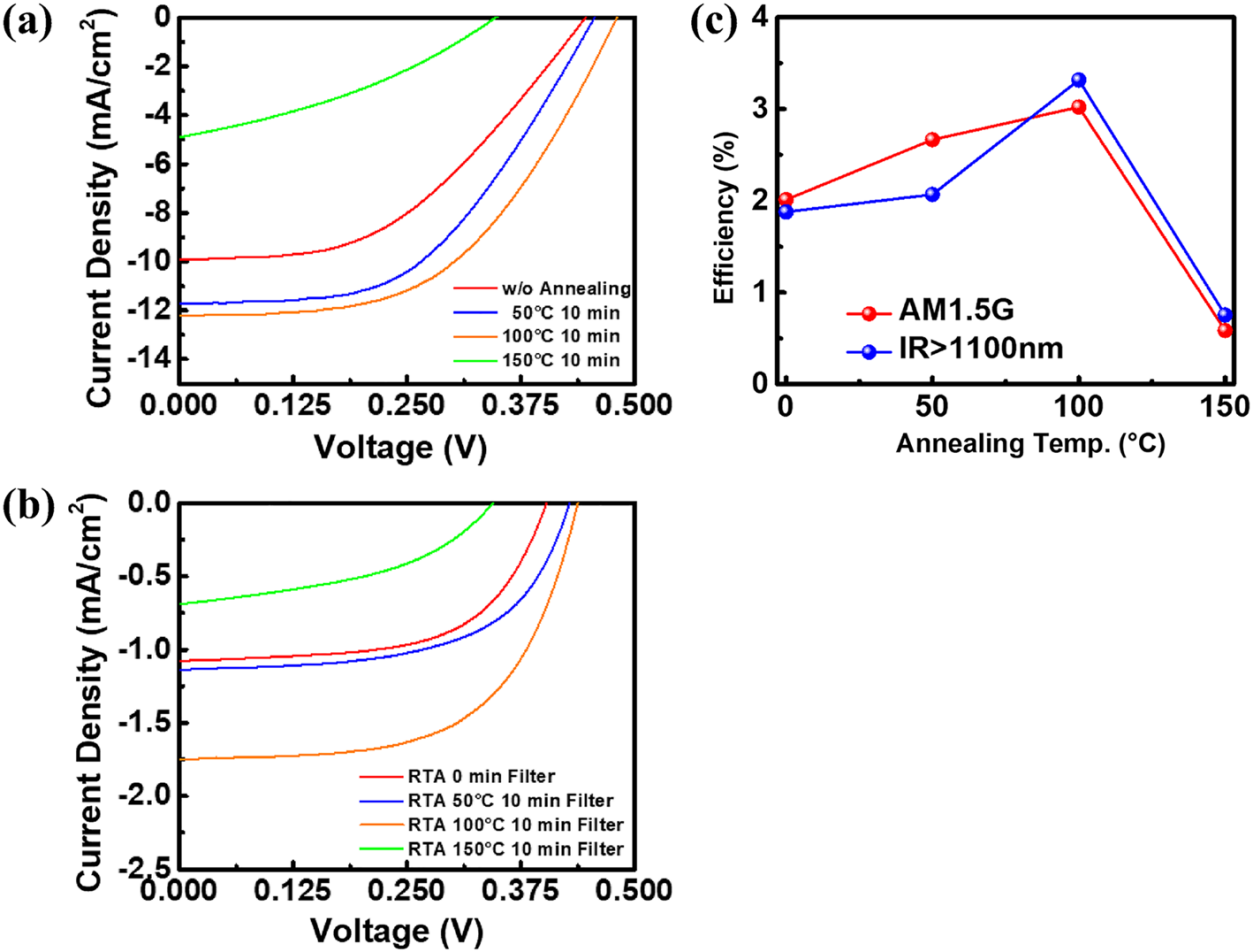
J–V curves of metal/silicon devices with different post-annealing temperature (**a**) with filter and (**b**) without filter, and (**c**) the change trend of photovoltaic conversion efficiency against temperature.

To examine the conversion efficiency of the device under different annealing temperatures to the whole solar spectrum, the J-V curve was first measured under AM1.5G with entire solar spectrum. The results are shown in Fig. 7a. With the increase of annealing temperature, the short circuit current J_sc_ and open circuit voltage V_oc_ increases due to the higher film conductivity and light absorptivity. The fill factor increases from 45.53% to 51.51% at 100 °C. Therefore, the solar efficiency is gradually improved. The highest efficiency is 3.022% under the annealing condition of 100 °C for 10 min. However, when the temperature is raised to 150 °C, the conversion efficiency dropped rapidly to 0.586% due to the decline of conductivity of the metal thin film. From the surface SEM in Fig. 6a–c, it can be seen that the silver film on the silicon substrate tends to gather locally to form a larger island structure under higher temperature. Cracks are preferentially found at the initial film defects during the aggregation process. The higher the degree of aggregation, the wider the crack is. Finally, there are many discontinuous metal islands. The discontinuous film structure makes carriers difficult to transport to the finger electrode. Hence, the photocurrent generated at 150 °C is only a quarter of which at 100° C and the solar efficiency reduced dramatically. The devices were then measured with infrared spectrum above 1100 nm. The measured J-V curve results are shown in Fig. 7b. All photovoltaic characteristics of hot-carrier photovoltaic conversion Schottky devices with different annealing temperature under different solar spectrum are listed in Table 2. The efficiency change trend is illustrated in Fig. 7c. The measurements under AM1.5G are plotted with the red line, while those under infrared above 1100 nm are with the blue line. Both red and blue lines show similar variation of conversion efficiency with annealing temperature. The highest efficiency is 3.316% at 100 °C for the incident light intensity of 13.85 mW/cm^2^. When the thermal annealing temperature continues to increase to 150 °C, the distance between silver islands increases, the conductivity of film declines, and then the efficiency drops rapidly to 0.755%.

**Table 2:** The photovoltaic characteristics of hot-carrier photovoltaic conversion Schottky devices with different annealing temperature under different solar spectrum.

Condition	Voc (V)	Jsc (mA/cm^2^)	Fill Factor (%)	Solar eff. (%)
Without filter				
w/o Annealing	0.446	9.91	45.53	2.012
50 °C 10 min	0.456	11.73	49.86	2.666
100 °C 10 min	0.480	12.21	51.51	3.022
150 °C 10 min	0.347	4.901	34.45	0.586
With filter				
w/o Annealing	0.403	1.077	59.88	1.878
50 °C 10 min	0.429	1.136	58.82	2.068
100 °C 10 min	0.438	1.747	60.04	3.316
150 °C 10 min	0.344	0.689	44.11	0.755

**Figure 8:**
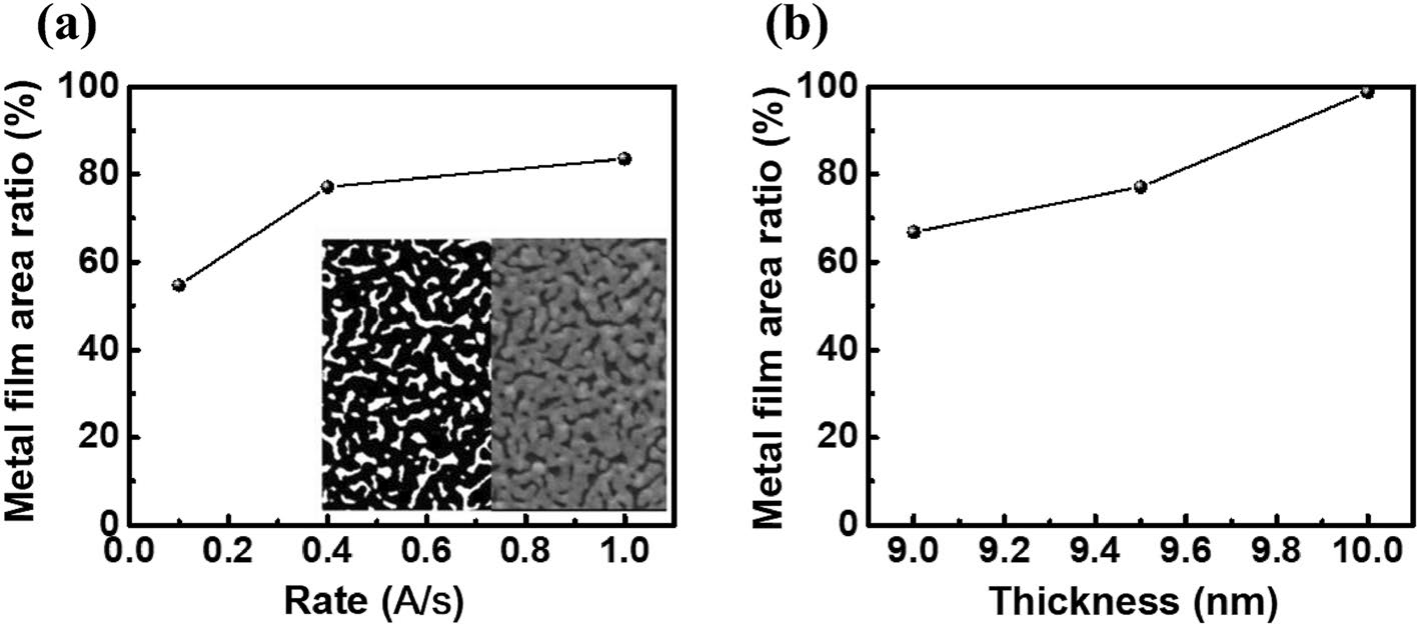
Effective area ratio of silver metal surface (**a**) changes with evaporation rate or (**b**) film thickness, and the effect of metal Island spacing on carrier tunneling probability.

The type of metal film affects the effective resistance and light absorptivity of the device in macroscopic view, and the distribution structure of metal particles affects the generation mechanism of hot carriers in microscopic view. Metal–silicon junction devices rely on the Schottky layer of the metal–semiconductor junction to absorb near-infrared light higher than the energy level of the silicon energy gap. The SEM images tells us that the silver film does not completely cover the entire surface of the silicon substrate on the scale of hundreds of nanometers, so the effective area is not consistent with the device area at the microscopic level, and it shows a trend with the change of evaporation rate and thickness. As shown in Fig. 8a,b, the distribution of the metal film was analyzed by software ImageJ, as well as with the increase of evaporation rate or thickness. The device with a 9.5-nm evaporation rate of 0.1 Å/s has the lowest conversion efficiency, and its metal film coverage rate is only 54.7%. When the deposition rate is increased to 0.4 Å/s and 1.0 Å/s, the coverage rate is increased to 77.2% and 83.6%, respectively. If the deposition thickness is increased to 10 nm at 0.4 Å/s, the coverage rate can reach nearly 98.6%. The film with high uniformity is beneficial to improve the effective area of the device to generate hot carriers.

From the electrical properties aspect, the metal-distribution density is closely related to the ability of the device to transfer current to the surface. It can be seen from the SEM that the growth of the metal thin film under the condition of a low-evaporation rate tends to be island nucleation instead of island expansion^40^. Thus the number of islands per unit area is large and the size is small. Also the islands are not connected with each other, limiting the conduction of carriers in the metal thin film. Besides, the tunneling probability^41^ is extremely sensitive to the distance between islands. As the distance increases, the tunneling probability decreases exponentially and significantly impacts the output photocurrent of the device. The experimental results also show that the conversion efficiency of the device under the lowest evaporation rate is less than 1% and less than half of the efficiency of the device with high-evaporation rate.

**Figure 9:**
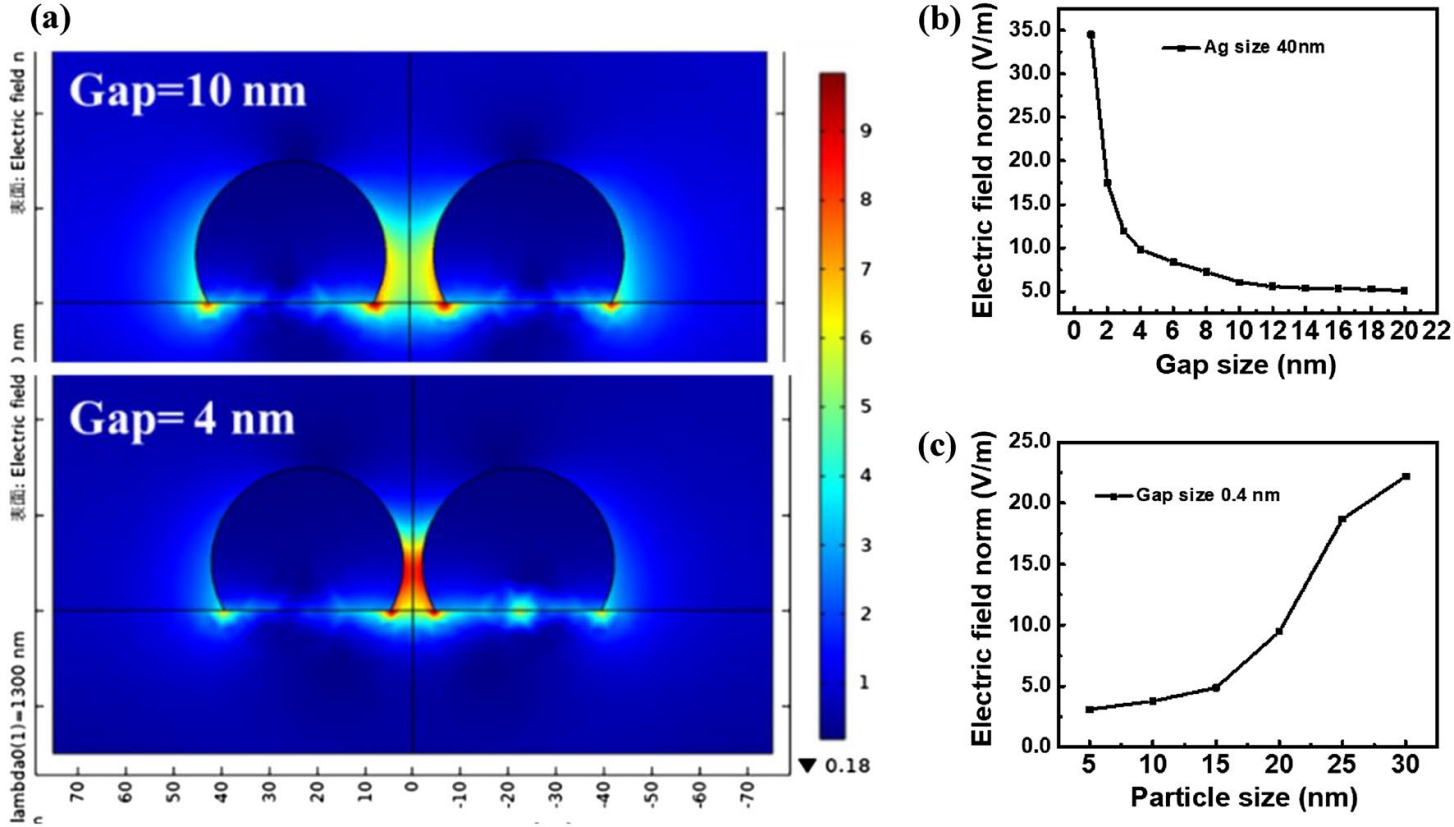
COMSOL numerical simulation of surface near-field distribution of silver metal particles with fixed particle size on silicon substrate at different pitches (**a**) 4 nm and 10 nm for incident light with wavelength of 1300 nm, and (**b**) fixed particle size, pitch change and (**c**) fixed pitch, varying particle size.

The high-conversion efficiency of the hot-carrier photovoltaic conversion Schottky device for infrared light comes from the interaction of the nanoparticles of the metal thin film and the light field, which causes the metal surface plasmon resonance, and thus improves the generation of hot carriers. It is known from the previous studies on the application of metal to plasma production that^35,36^ the size and distribution density of nanoparticles in metal thin films are important factors in determining the excitation of hot carriers by plasma. The incident light field induces the free electron group on the surface of metal particles to generate a harmonic oscillation perpendicular to the surface, and the surface plasmon resonance effect strengthens the interaction between light and matter. Also, the near-field intensity of the induced concentrated metal surface promotes the absorption of light by the thin film^43^. We simulated the surface near-field distribution of silver particles with different distribution conditions on the silicon substrate under the incident light with a wavelength of 1300 nm through the software COMSOL. As shown in Fig. 9a, we set the silver particle size of 40 nm and the particle spacing of 4 nm and 10 nm, respectively. It is observed that the surface electric field distribution with small distribution spacing of metal particles is highly concentrated at the island-like gaps, and the maximum electric field of 4 nm spacing is more than 4 times that of 10 nm spacing. We investigated the change of the spacing between the 2 particles with the particle size being fixed, and summarized the electric field calculation results to obtain Fig. 9b. The electric field amplification ratio is extremely sensitive to the spacing between the metal particles. When the spacing is reduced from 10 to 1 nm, the maximum electric field increases from 6.06 V/m to 34.5 V/m, which is nearly 570%, so the intensity of light increases about 32 times. Next, we discuss the influence of particle size on the electric field. Under the condition of maintaining the spacing, the particle size is changed. The change of electric field is shown in Fig. 9c. When the particle size increases from 5 to 25 nm, the electric field increases for about 7 times and the intensity of light increases for about 49 times. This is because the arc diameter of the reflecting surface of the resonant cavity increased, which improved the light-capturing ability of the thin film. The enhanced intensity of light from the nano-structures of metal indicates that the hot-carrier concentration is significantly increased locally and could lead to much faster diffusion toward the metal-Si junction for less energy loss to transport over the energy barrier. From the simulation results, the metal thin film under a high-evaporation rate has advantages such as high uniformity and conductivity. Meanwhile, the silver metal thin film with large particle size and high-distribution density can more effectively induce surface plasmon resonance and further improve the absorption of infrared light by the device. It can be seen that the metal thin film type has a great impact on the hot-carrier photovoltaic conversion Schottky devices.

**Figure 10:**
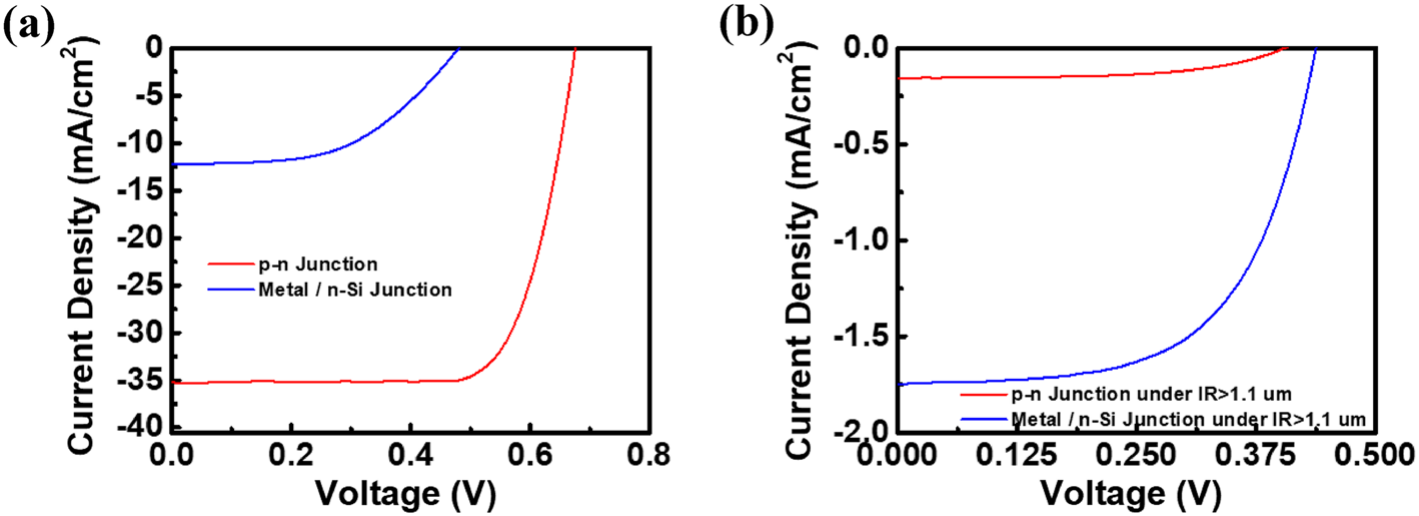
J–V curve of metal/silicon junction and silicon p–n junction solar cells under (**a**) AM 1.5G solar spectrum and (**b**) infrared regime above 1100 nm.

Finally, from the previous series of tests of different evaporation rates and thicknesses, according to the conversion efficiency, the best efficiency was measured under the parameters of an evaporation rate of 0.4 Å/s and thickness of 9.5 nm. The self-made hot-carrier photovoltaic conversion Schottky device was compared with the commercially available silicon p–n junction solar cells. If the Si filter is not used, the efficiency of the silicon p–n junction cell under illumination is 17.67%. The measurement result is represented as a red line in Fig. 10a, indicating that its optical power-conversion function is normal. Then, the metal–silicon junction device of our team was used for the same measurement, and its conversion efficiency is 3.022%; the measurement result is represented as a blue line in Fig. 10a. Its optical power-conversion effect is worse than that of silicon p–n junction solar cells. However, when we use a Si filter to block the spectrum below 1100 nm, the incident light cannot be absorbed by the single silicon p–n junction solar cell, and its conversion efficiency is almost 0. In contrast, the measured efficiency of the hot-carrier photovoltaic conversion Schottky device is 3.316%. The measurement results are shown in Fig. 10b. All photovoltaic characteristics of different solar cells under different solar spectrum are listed in Table 3. Compared with the commercially available silicon solar cells, the hot-carrier photovoltaic conversion Schottky device produced by our laboratory shows effective optical- to electrical-conversion ability for a wavelength above 1.1 microns, which is helpful to the utilization of the whole solar spectrum.

**Table 3:** The photovoltaic characteristics of different solar cells under different solar spectrum.

Device	Voc (V)	Jsc (mA/cm^2^)	Fill Factor (%)	Solar eff. (%)
Under AM 1.5G solar spectrum				
p–n junction	0.676	35.21	74.28	17.67
metal/n-Silicon	0.480	12.21	51.51	3.022
Under infrared regime above 1100 nm				
p–n junction	0.404	0.155	56.44	0.256
metal/n-Silicon	0.438	1.747	60.04	3.316

## Conclusion

In this study, we achieved the effect of expanding the absorption spectrum range of silicon-based solar devices through a simple and low-cost process, as well as control the deposition of silver metal films as light-absorbing layers on silicon substrates by adjusting the evaporation rate, thickness parameters, and post-annealing process. When the evaporation rate is higher, the silver film is more uniform on the substrate and then causes higher carrier-collection capacity in addition to lower absorptivity. The fine adjustment of thickness can control the gap width between silver film islands and the island size, further affecting the conductivity, light-catching ability, coverage area, and ability of exciting and deriving hot carriers of the thin film. The post-annealing can improve the crystallinity of the silver film and the conductivity of the device. In terms of the photoelectric efficiency, a 9.5-nm silver film was deposited at the rate of 0.4 Å/s, and the best conversion efficiency was measured to be 3.316% under the infrared spectrum with a wavelength longer than 1.1 μm. A hot-carrier photovoltaic conversion Schottky device that can effectively absorb the infrared spectrum of more than 1100 nm was successfully made. It provides the promise for the realization of full-spectrum solar cells through the hot-carrier effect.

## Methods

The silicon substrate was cleaned with standard cleaning procedures (Type: n, orientation: 1–0–0, resistance: 2–7 Ω cm, thickness: 600–610 μm, size: 2.5 cm × 2.5 cm) to remove the particles and dirt. A nitrogen gun was used to quickly volatilize the residual organic solvent, and then the cleaned substrate was soaked in a buffer oxide etching solution (BOE) to remove the oxides naturally generated on the substrate surface. After cleaning the silicon substrate, a 9.5-nm-thick silver film was deposited under atmospheric pressure of 4 × 10^−6^ Torr through the thermal-evaporation process. We tested the effect of different evaporation rates (0.1–1.0 Å/s) during the deposition of silver film, and then we deposited a 100-nm silver finger electrode and aluminum back electrode onto the metal layer and the other side of the substrate, respectively. The best conversion efficiency was obtained from the measurement of components with different evaporation rate parameters. With the rate of the former best results, we deposited metal layers with different thicknesses (9–10 nm) and tested the influence on metal layer appearance and solar efficiency.

## Data Availability

The datasets used and/or analyzed during the current study available from the corresponding author on reasonable request.
